# PET microplastics induce lipotoxicity in the porcine pancreas

**DOI:** 10.1186/s12864-025-12507-8

**Published:** 2026-01-07

**Authors:** Karol Mierzejewski, Aleksandra Kurzyńska, Monika Golubska, Robert Stryiński, Ismena Gałęcka, Jarosław Całka, Paula Borrajo, Manuel Pazos, Mónica Carrera, Iwona Bogacka

**Affiliations:** 1 Department of Animal Anatomy and Physiology, University of Warmia and Mazury in Olsztyn, Oczapowskiego 1a, Olsztyn, 10-719 Poland; 2 Department of Biochemistry, University of Warmia and Mazury in Olsztyn, Olsztyn, Poland; 3 Department of Epizootiology, University of Warmia and Mazury in Olsztyn, Olsztyn, Poland; 4 Department of Clinical Physiology, University of Warmia and Mazury in Olsztyn, Olsztyn, Poland; 5 Department of Food Technology, Institute of Marine Research (IIM), Spanish National Research Council (CSIC), Vigo, Spain

**Keywords:** Pancreas, Fatty acid, Trypsinogen, Polyethylene terephthalate

## Abstract

Microplastics are a relatively new discovered environmental hazard that can contribute to the disruption of many physiological processes in the organism. There is evidence that they affect the physiology of the pancreas, but research in this area remains limited. Therefore, the aim of the study was to determine the effects of PET microplastics on the global proteomic profile of the pancreas using LC–MS/MS analysis, with the pig as a model organism. The pigs were treated either with a low (0.1 g/day) or a high dose (1 g/day) of PET microplastics for four weeks. The analysis revealed that PET microplastics affected protein abundance in a dose-dependent manner – the low dose altered the abundance of 7 proteins, while the high dose – 17. The differentially regulated proteins were involved in fatty acid biosynthesis (FASN, KAS), lipid peroxidation (CBR1) and digestive enzyme production (trypsinogen). Complementary colorimetric assays confirmed a significant increase in free fatty acids under the influence of a high dose of PET microplastics. Taken together, these results indicate that PET microplastics may affect oxidative status, induce lipotoxic stress and impair pancreatic exocrine function, suggesting a novel pathway through which microplastics may cause metabolic disturbances.

**Supplementary Information:**

The online version contains supplementary material available at 10.1186/s12864-025-12507-8.

## Introduction

Increasing production of plastic during last decades has caused microplastics (MPs) contamination in all environments including soils, water and air [1]. Microplastics are defined as small plastic particles (less than 5 mm) generated from the breakdown of larger plastic items or produced intentionally as primary microplastics [2]. Humans are exposed to MPs mainly through ingestion, where the ingested particles can undergo gastrointestinal modification, cross the intestinal barrier, and translocate to distant organs [3–5]. Evidence of MPs in human and animal tissues has raised concerns regarding their potential systemic effects, particularly on metabolically active organs, as experimental studies have shown that MPs can induce oxidative stress, DNA damage, and disruptions of molecular pathways [6].

Among the various plastic types, polyethylene terephthalate (PET) is particularly important due to its widespread use in food and beverage packaging, making it one of the major sources of microplastic intake through the diet [7]. Recent studies have detected PET particles in human blood and feces, demonstrating that ingested PET microplastics can cross the gastrointestinal barrier and undergo systemic distribution under real-life exposure conditions [8, 9]. Our previous studies have also demonstrated that PET MPs affected the expression of miRNAs and metabolites involved in pancreatic carcinogenesis and metabolic disorders in serum-derived extracellular vesicles (EVs), induced oxidative stress, and altered the expression of inflammatory in the pancreas [10–12], as well as increased serum insulin levels and glucose in pancreatic tissue [13].

Over the past two decades, the global incidence of pancreatitis has increased by almost 60%, with high-income regions bearing the greatest burden [14]. Furthermore, the risk of developing diabetes mellitus after acute pancreatitis (AP) is significant. Approximately 20% of patients develop new-onset diabetes within five years of their first AP episode, and this number can increase to approximately 40% with longer follow-up [15]. While MPs from several polymers, such as PVC and polystyrene, have been shown to induce pancreatic inflammation, fibrosis, metabolic dysregulation, and insulin resistance in animal models [16–18], the biological impact of PET remains far less understood. Therefore, the aim of this study was to determine the effect of PET on global proteomic profile of pancreas by tandem mass tag (TMT)-quantitative proteomics and LC–MS/MS analysis using the pig as a model.

The pig is widely recognized as a valuable model organism due to its close anatomical, physiological, immunological, and metabolic similarity to humans [19]. These similarities are particularly evident in the gastrointestinal tract, including pancreatic structure, endocrine and exocrine function, and glucose–insulin regulation, making pigs highly suitable for studying metabolic diseases [20]. Importantly, pigs are also considered one of the most relevant large animal models in toxicology, with strong predictive value for human toxicodynamic and toxicokinetic responses [21]. In addition, we used immature animals, as developing organisms are generally more susceptible to the adverse effects of toxic substances. Notably, infants have been estimated to experience significantly higher daily exposure to PET microplastics than adults [21], and early-life exposure may disrupt physiological regulatory processes later in life [22]. Therefore, the use of immature pigs provides a relevant model for assessing the potential developmental metabolic effects of microplastic exposure.

## Materials and methods

### Animals

All experimental procedures were approved by the Local Ethics Committee of the University of Warmia and Mazury in Olsztyn (Approval No. 10/2020, issued February 26, 2020) and followed Polish and EU regulations on the use of animals in research (Directive 2010/63/EU). Experimental design and reporting conformed to the ARRIVE guidelines. The study was conducted on 15 eight-week-old female pigs (Pietrain × Duroc; ~ 20 kg) obtained from a commercial breeder in Lubawa, Poland. The animals were housed under controlled environmental conditions (temperature 20–22 °C, humidity 55–60%), with free access to water and a nutritionally balanced diet. To avoid contamination, all feeding and watering systems were made of stainless steel, and bedding consisted of wood shavings. Animals were randomly divided into three groups (*n* = 5 per group): a control group (CTR) receiving empty gelatin capsules, a low-dose group (LD) given 0.1 g of PET microplastics per pig per day, and a high-dose group (HD) receiving 1 g/pig/day. Capsules were administered orally one hour before the morning meal. The PET microplastics (semi-crystalline powder; Goodfellow Cambridge Ltd., UK, catalog no. ES306031/1) had particle sizes ranging from 7.6 µm to 416.9 µm, predominantly around 158.5 µm, and varied in shape (spherical, fibrous, irregular), as described previously [23]. The doses were chosen based on estimates of human exposure to microplastics (0.1–5 g per week [3]. After four weeks of exposure, all animals were euthanized humanely. Sedation was achieved by intramuscular administration of atropine (0.05 mg/kg, Polfa, Poland), xylazine (3 mg/kg, Vet-Agro, Poland), and ketamine (6 mg/kg, Vetoquinol Biowet, Poland). Deep anesthesia was confirmed about 20 min post-injection, followed by intravenous administration of sodium pentobarbital (0.6 mL/kg, Biowet, Poland) to induce euthanasia. Immediately after the death was confirmed, pancreatic tissue was excised, rinsed in ice-cold PBS to remove blood, frozen in liquid nitrogen, and stored at − 80 °C until further proteomic and biochemical analyses.

### Protein extraction

Total protein was isolated from pancreatic tissue collected from pigs exposed to low (LD PET; *n* = 5) or high (HD PET; *n* = 5) doses of PET microplastics, and from the control group (CTR; *n* = 5). Approximately 30 mg of tissue was homogenized in lysis buffer and processed as previously described [24]. Protein concentration was measured using the BCA assay. For proteomic analysis, 100 µg of protein from each sample was precipitated with cold acetone and subjected to tryptic digestion following our established protocol [25, 26].

### TMT labeling and high-pH reversed-phase fractionation

Peptide labeling was performed using TMT 16-plex reagents (Thermo Fisher Scientific) according to the manufacturer’s instructions and our previously described protocol [25]. Labeling was performed using the following TMT reporter tags: control group (CTR) – 126, 127 N, 127 C, 128 N, 128 C; low-dose PET (LD PET) – 129 N, 129 C, 130 N, 130 C, 131 N; high-dose PET (HD PET) – 131 C, 132 N, 132 C, 133 N, 133C. After labeling, all samples were combined in equal amounts to generate a single multiplexed sample. To improve peptide coverage, the pooled material was fractionated using high-pH reversed-phase chromatography (Pierce kit), following the supplier’s recommendations. The resulting fractions were dried and stored at − 80 °C until LC–MS/MS analysis.

### LC–MS/MS analysis and data processing

Peptide fractions were analyzed on a Vanquish Neo UHPLC system coupled to an Orbitrap Eclipse mass spectrometer equipped with a FAIMS Pro interface (Thermo Fisher Scientific). Peptides were separated by nano-flow reverse-phase chromatography on an EASY-Spray C18 analytical column, and data were acquired in positive ion mode using a data-dependent acquisition (DDA) strategy combined with FAIMS-based ion selection. Each fraction was injected in duplicate to provide technical replicates. Raw MS data were processed in Proteome Discoverer 3.1 using the SEQUEST HT algorithm. Spectra were searched against the *Sus scrofa* UniProt/TrEMBL database with standard settings for TMT-based proteomics: full tryptic specificity (up to two missed cleavages), 10 ppm precursor and 0.06 Da fragment mass tolerances, fixed TMT and carbamidomethylation modifications, and variable oxidation and N-terminal acetylation. Peptide-spectrum matches were validated with Percolator at a 1% FDR threshold.

### Statistical analysis of proteomics data and visualizations

Statistical evaluation of peptide identifications was performed using the Percolator node implemented in Proteome Discoverer 3.1 (Thermo Fisher Scientific, Waltham, MA, USA), applying a false discovery rate (FDR) cutoff below 1%. Only master proteins quantified with at least one unique peptide and detected across all experimental groups were retained for downstream analyses. Relative quantification was carried out with normalization to the total peptide amount. For relative protein abundance determination in each sample, the Minora Feature Detector node was applied, followed by the “ANOVA (individual proteins)” node in Proteome Discoverer 2.4 (Thermo Fisher Scientific). The ANOVA *p*-values reported correspond to the statistical calculations performed internally by Proteome Discoverer, and no additional multiple-testing correction was applied. Proteins were considered differentially regulated if they displayed a minimum 1.3-fold change in normalized abundance and reached statistical significance. Heatmaps illustrating expression profiles were generated using the pheatmap package in R. To visualize global protein abundance changes, volcano plots were produced in Python using Plotly Express, Pandas, and NumPy. The x-axis represents log_2_ (fold change) and the y-axis − log_10_ (*p*-value). Proteins meeting both statistical and fold-change thresholds were highlighted as significantly regulated (up-regulated = red, down-regulated = green), while nonsignificant entries were shown in gray. Thresholds were indicated by dashed lines. Comparative volcano plots (HD PET vs. CTR and LD PET vs. CTR) were further assessed using Pearson’s correlation coefficient to identify proteins showing inverse abundance trends (“shifted” proteins).

### Functional categories of identified proteins

The final list of DRPs obtained in each experiment was classified into three Gene Ontology (GO) categories: biological processes, cellular components, and molecular functions. Identified proteins were also assigned to the metabolic pathways in which they are involved. The analyses were performed using two R packages dedicated to GO and pathway enrichment analysis: clusterProfiler [27] and Pathview [28]. Statistical significance was set at *p* ≤ 0.05.

### Protein–protein interactions network analysis

Network analysis was performed by submitting the DRPs dataset to Cytoscape (v. 3.8.0; NIGMS, Bethesda, MD, USA), a software platform for visualizing complex networks, and analyzing it with stringApp (v. 1.5.1) [29]. Protein interactions were identified by comparing the input data with the *Sus scrofa* background. All interactions were visualized based on co-expression, co-occurrence and the presence of information on protein interactions in different databases. The Markov clustering algorithm (MCL) was used to cluster the obtained networks (inflation parameter = 3).

### Fatty acid synthase activity assay

FASN activity was measured using the CheKine™ Micro FASN Activity Assay Kit (Abbkine, KTB2240) following the manufacturer’s protocol. Briefly, pancreatic tissue was homogenized in extraction buffer, and clarified supernatants were used for analysis. Absorbance at 340 nm was recorded to monitor NADPH oxidation, and FASN activity was calculated according to the kit instructions and expressed as U/g protein. Protein concentrations were determined separately using the BCA assay.

### Non-esterified/free fatty acids (NEFA) assay

NEFA levels in pancreatic tissue were measured using the NEFA/FFA Colorimetric Assay Kit (Thermo Fisher Scientific, EEA017) according to the manufacturer’s instructions. Briefly, homogenized tissue supernatants were processed following the kit protocol, and absorbance was recorded at 715 nm. NEFA concentrations were calculated from a palmitic acid standard curve and expressed as μmol/g of tissue.

## Results

### PET treatment lead to changes in the proteome of pancreas

**Fig. 1:**
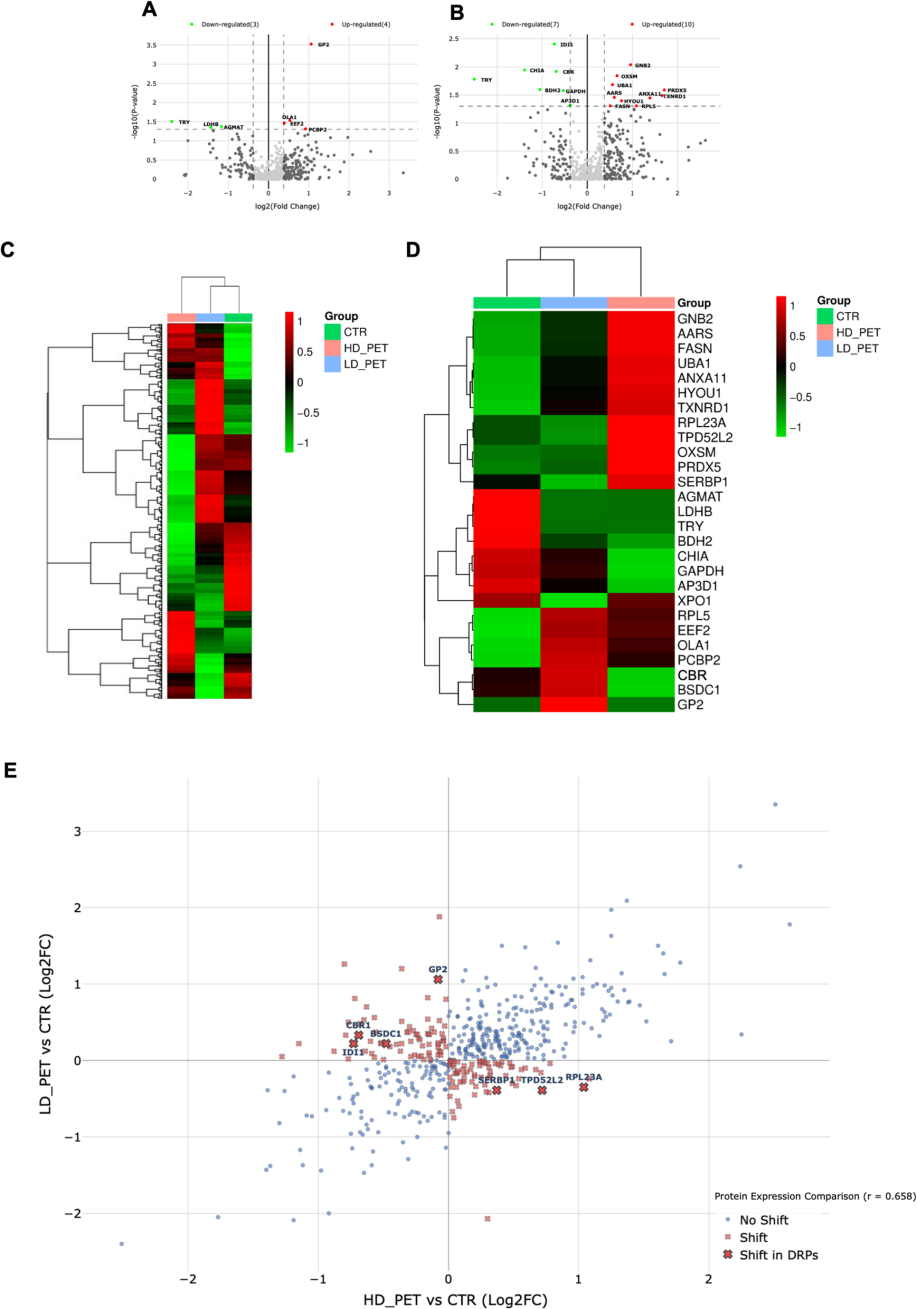
Differentially regulated proteins (DRPs) identified in the porcine pancreas after treatment with PET. Volcano-plot representations of statistical analysis of performed comparisons: (**A**) low dose of PET (LD PET) vs. control (CTR), (**B**) high dose of PET (HD PET) vs. CTR (FC ≥ 1.3, *p*-value ≤ 0.05). In presented volcano plots, the most upregulated proteins are towards the right (red), the most downregulated proteins are towards the left (green), and out of them the most statistically significant proteins were towards the top. Heatmap of the general proteome (**C**) and only identified DRPs (**D**) in three tested groups (CTR vs. two PET doses). Red (upregulated) and green (downregulated) squares describe abundance in the compared groups. Scale on the figure. **E** The scatter plot showing the protein abundance shifts using the relationship between Log2FC in the HD PET vs. CTR comparison and Log2FC in the LD PET vs. CTR comparison. Protein abundance shifts were categorized based on directional changes, where proteins with opposite abundance patterns (positive in one condition and negative in the other) were classified as “shifted”. Color coding was added to the scatter plot to distinguish between regular proteins (blue circles), shifted proteins (red X) and shifted proteins in the DRP group (red bold X with names). All fold change (FC) values were expressed as log2 values. Detailed information about the visualized data can be found in the Supplementary Tables 1–4

As a result of LC–MS/MS analysis, we identified a total of 13,069 proteins (Supplementary Table 1). The data were visualized on Volcano plots (Fig. 1). The proteome of the pancreas has been slightly altered when treated with LD PET (Fig. 1A). Four proteins were found to be upregulated, and 3 proteins were downregulated compared to pancreas without PET treatment (FC ≥ 1.3; *p* ≤ 0.05; Supplementary Table 2). More profound changes were observed in the proteome of the pancreas when treated with HD PET (Fig. 1B). Proteomic analysis showed that 17 proteins were differentially regulated compared to the control, with 10 proteins being upregulated and 7 proteins being downregulated (FC ≥ 1.3; *p* ≤ 0.05; Supplementary Table 3). To show the general pattern of protein regulation under the PET treatment the heatmaps were created (Fig. 1C, D). In the Fig. 1E only DRPs were shown. The analysis of distribution of common and unique DRPs after two different treatments showed that one protein – trypsinogen (TRY) – was affected by PET in both doses. To investigate if DRPs exhibit different patterns of abundance in comparisons between two PET doses and control, the scatter plot was created (Fig. 1E). Seven DRPs were found to have opposite abundance patterns in LD PET treatment and HD PET treatment (RPL23A, IDI1, TPD52L2, CBR1, BSDC1, SERBP1, GP2), what means that these DRPs abundance was downregulated in one condition and upregulated in the other (Supplementary Table 4).

### DRPs are associated with diverse biological functions and pathways in pancreas

**Fig. 2:**
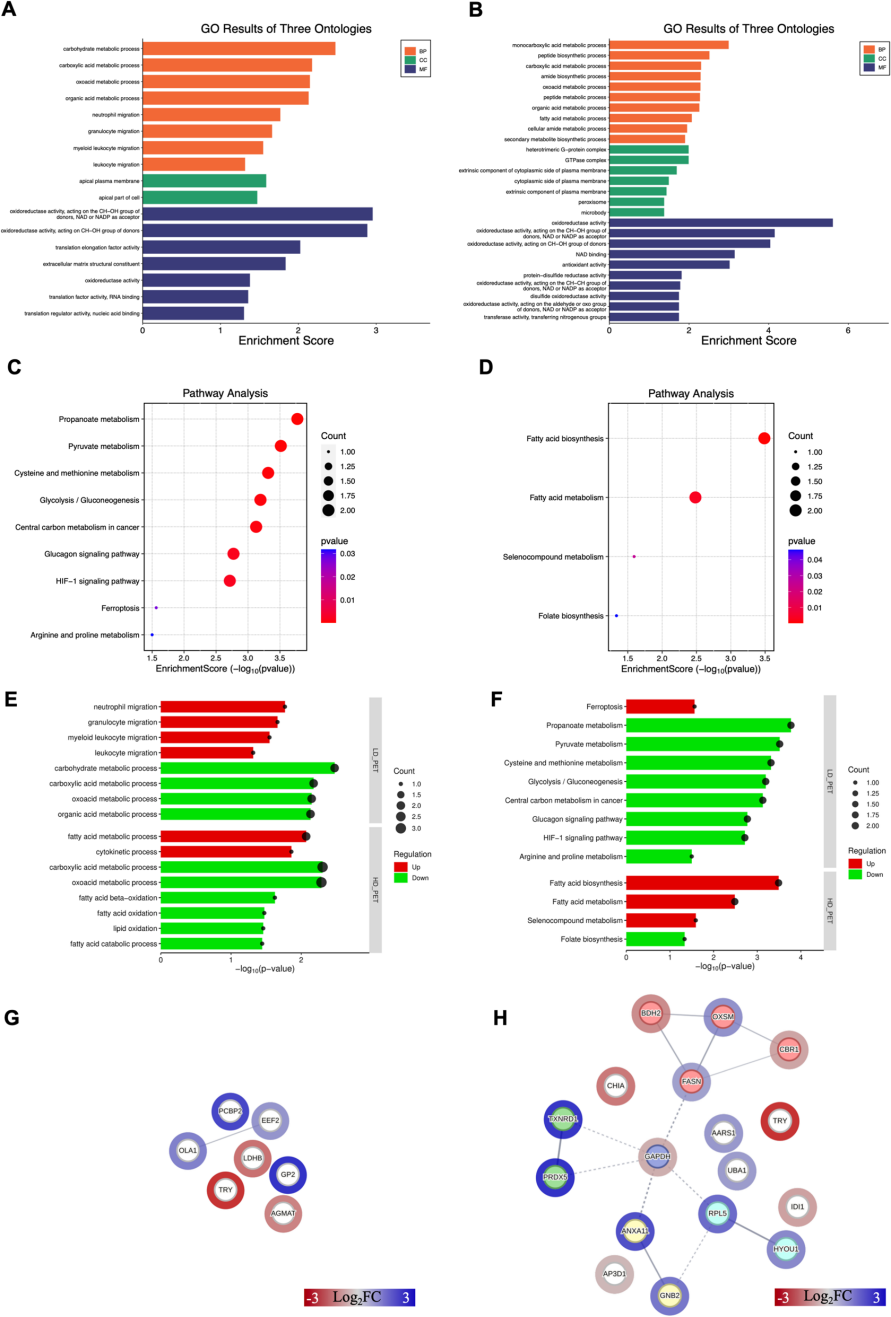
Functional characterization of the differentially regulated proteins (DRPs) identified in the pancreas of pigs after treatment with PET. The visualization of GO enrichment analysis results of DRPs after treatment with a low dose of PET (LD PET) (**A**) and high dose of PET (HD PET) (**B**). Pathways enrichment analysis results for LD PET and HD PET are visualized in part (**C**) and (**D**), respectively. Selected enriched biological processes (**E**) and metabolic pathways (**F**), identified under each treatment condition, are categorized according to their direction of regulation. Upregulation – red and downregulation – green. Interactions between DRPs identified in pancreas after treatment with a LD PET (**G**) and HD PET (**H**). Red circles – up-, green circles – downregulated proteins. Scale on the figure. Detailed information about the visualized data can be found in the Supplementary Tables 5–11

Differentially regulated proteins (DRPs) identified in the pancreas of pigs after treatment with a LD PET or HD PET of PET were subjected to Gene Ontology (GO) and KEGG pathway enrichment analysis. GO analysis of DRPs in the LD PET group revealed statistically significant enrichment (*p* ≤ 0.05) in 8 biological processes (BP), 2 cellular components (CC), and 7 molecular functions (MF) (Fig. 2A, Supplementary Table 5). The most prominent BP terms included carbohydrate metabolic process (GO:0005975), carboxylic acid metabolic process (GO:0019752), and oxoacid metabolic process (GO:0043436), all associated with L-lactate dehydrogenase (LDHB). Proteins were localized to CC terms including apical plasma membrane (GO:0016324) and apical part of cell (GO:0045177). The MF terms included, among others, oxidoreductase activity (GO:0016616, GO:0016614, GO:0016491), translation elongation factor activity (GO:0003746), and nucleic acid binding activity (GO:0008135, GO:0090079). In the HD PET group, GO enrichment analysis revealed associations with 33 biological processes (BP), 7 cellular components (CC), and 24 molecular functions (MF) (Fig. 2B, Supplementary Table 6). The enriched BP terms included monocarboxylic acid metabolic process (GO:0032787), fatty acid metabolic process (GO:0006631), and fatty acid beta-oxidation (GO:0006635). DRPs were localized to CC terms such as heterotrimeric G-protein complex (GO:0005834), GTPase complex (GO: 1905360), cytoplasmic side of plasma membrane (GO:0009898), and peroxisome (GO: 0005777). Enriched MF terms included oxidoreductase activity, acting on the CH-OH group of donors, NAD or NADP as acceptor (GO:0016616), NAD binding (GO:0051287), antioxidant activity (GO:0016209), and thiolester hydrolase activity (GO:0016790), with proteins such as fatty acid synthase (FASN), carbonyl reductase [NADPH] (CBR1), dehydrogenase/reductase SDR family member 6 (BDH2), and thioredoxin reductase 1 (TXNRD1). Pathway enrichment analysis of the LD PET group identified 9 significantly enriched KEGG pathways (Supplementary Table 7), including propanoate metabolism (ssc00640), pyruvate metabolism (ssc00620), glycolysis/gluconeogenesis (ssc00010) and glucagon signaling pathway (ssc04922), all involving LDHB (Fig. 2C). In the HD PET group, pathway analysis revealed 4 enriched KEGG pathways (Supplementary Table 8): fatty acid biosynthesis (ssc00061), fatty acid metabolism (ssc01212), selenocompound metabolism (ssc00450) and folate biosynthesis (ssc00790). These were linked to proteins such as 3-oxoacyl-[acyl-carrier-protein] synthase, mitochondrial (OXSM), FASN, CBR1 and TXNRD1 (Fig. 2D). Selected enriched biological processes and metabolic pathways, identified under each treatment condition, were categorized according to their direction of regulation (up- or downregulated) and are shown in Fig. 2E and F, respectively.

### DRPs establish a moderate network of interactions

The visualizations of protein interactions demonstrated week or moderately interacted networks (Fig. 2G, H; Supplementary Table 9). Seven DRPs in LD PET group, constituted a network with 1 interaction between Obg-like ATPase 1 (OLA1) and Tr-type G domain-containing protein (EEF1) (Fig. 2G). A total of 17 DRPs identified in HD PET group, constituted a network of intermediate connectivity, indicating a moderate degree of functional association among the identified proteins (14 interactions) (Fig. 2H). The proteins with the highest number of interactions with other proteins were glyceraldehyde-3-phosphate dehydrogenase (GAPDH; 5 interactions), FASN (4 interactions), OXSM (3 interactions) and large ribosomal subunit protein uL18 (RPL5; 3 interactions). MCL clustering revealed 5 clusters (Supplementary Table 10). These clusters included proteins involved in processes such as fatty acid biosynthesis and redox regulation (cluster no. 1; BDH2, CBR1, FASN, OXSM), signal transduction and membrane trafficking (cluster no. 2; ANXA11, GNB2), redox-active center (cluster no. 3; PRDX5, TXNRD1) or stress response and translational regulation (cluster no. 4; HYOU1, RPL5). The analysis classified GAPDH as a separate cluster no. 5 (Fig. 2H, Supplementary Table 11).

### FASN activity and FFA content

**Fig. 3:**
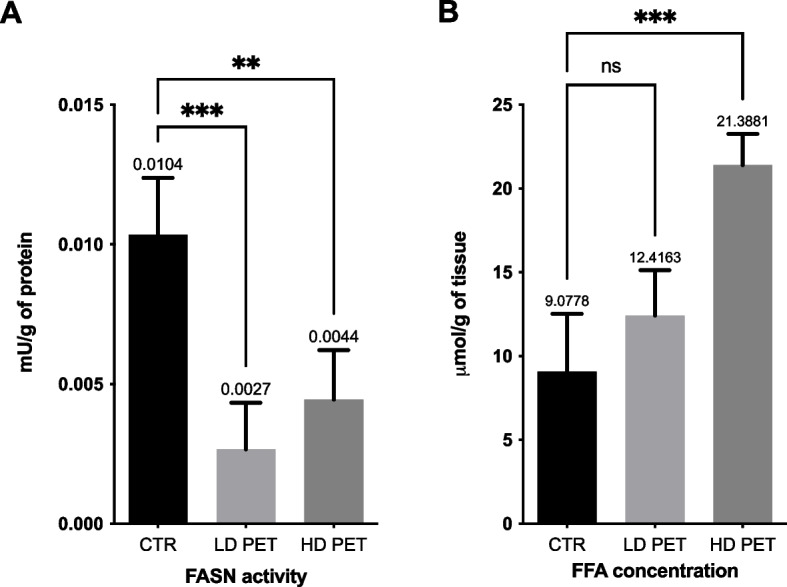
Effects of PET microplastics on pancreatic lipid metabolism. **A** FASN activity (mU/g protein) significantly decreased in both low dose of PET (LD PET) and high dose of PET (HD PET) groups vs. control (CTR). **B** Free fatty acid (FFA) levels (µmol/g tissue) increased after PET exposure, with a significant rise only in the HD PET group compared to CTR

To investigate the effects of PET microplastics on lipid metabolism in the pancreas, the activity of fatty acid synthase (FASN) and the concentration of free fatty acids (FFAs) were quantified. FASN activity (Fig. 3A) was significantly decreased in both PET-exposed groups compared to controls. Specifically, control animals exhibited a FASN activity of 0.0104 U/g protein, while the LD PET and HD PET groups showed reduced activities of 0.0027 U/g and 0.0044 U/g protein, respectively. The reduction was statistically significant for both LD PET (*p* < 0.001) and HD PET (*p* < 0.01) groups when compared to controls. In contrast, the concentration of FFAs showed a dose-dependent increase (Fig. 3B). The control group exhibited an average FFA concentration of 9.0778 μmol/g of tissue, which reached 12.4163 μmol/g in the LD PET group and 21.3881 μmol/g in the HD PET group. A statistically significant elevation was observed only in the HD PET group compared to the control (*p* < 0.001), while the change in the LD PET group was not statistically significant.

## Discussion

The presence of microplastics in the human organism and the associated effects have become an increasing concern as a growing body of research continues to elucidate the biological processes it disrupts. The current study provides evidence that PET microplastics interfere with porcine pancreatic physiology by modulating protein abundance. The differentially regulated proteins identified are primarily involved in metabolic pathways related to fatty acid synthesis, lipid and protein metabolism and oxidative stress. Although these findings come from a porcine model, the anatomical and physiological similarity to humans makes them potentially relevant for human health.

One of the first effects of microplastics on tissue is the triggering of oxidative stress and inflammation. This has been confirmed in various tissues, including our previous study on the pancreas, in which we showed that PET microplastics affect oxidative status in a dose-dependent manner [11, 30]. The current proteomic data showed that a high dose of PET MPs increased the abundance of peroxiredoxin 5 (PRDX5) and thioredoxin reductase 1 (TRX1), which are main antioxidant proteins. TRX1 acts against a variety of ROS such as singlet oxygen, hydroxyl radicals and H_2_O_2_ and has anti-inflammatory properties by suppressing the infiltration at the site of inflammation [31, 32]. Other studies also point to its protective role in the development of acute pancreatitis [33]. In addition, a higher abundance of different isoforms of peroxiredoxins and thioredoxin reductase in β-cells supports their function and survival in response to a variety of oxidative stressors [34]. It has also been reported that PRDX5 is activated in response to high glucose levels in cells [35]. Glucose is known to increase ROS production and cytotoxicity, while PRDX5 appears to play a role in the detoxification of excess H_2_O_2_ and the protection of cellular organelles. Our previous study has shown that a high dose of PET microplastics increases glucose levels in the pancreas. Therefore, we postulate that the increased abundance of TRX1 and PRDX5 in the pancreas may be a protective mechanism in response to PET microplastics.

Our study revealed that a high dose of PET microplastics decreases carbonyl reductase 1 (CBR1) – an NADPH-dependent enzyme belonging to a family of short-chain dehydrogenases/reductases [36]. CBR1 plays a key role in redox processes by reducing quinones such as ubiquinone-1 and tocopherolquinone, thus protecting biological membranes from lipid peroxidation [37]. In addition, CBR1 neutralizes reactive lipid aldehydes and alleviates oxidative stress, suggesting its crucial role in cellular protection against oxidative damage [38]. Decreased CBR1 abundance also leads to the accumulation of reactive 4-HNE – a product of polyunsaturated fatty acid peroxidation that damages proteins, lipids and DNA [39]. CBR1 is known to convert 4-HNE into the less reactive 1,4-nonanediol and thus inhibits the further peroxidative cascade [40]. Our study suggests that the decrease in CBR1 under the influence of a high dose of PET microplastics may contribute to the loss of an important mechanism for reducing reactive ketones and lipid aldehydes, which not only increases lipid peroxidation but also impairs redox homeostasis. The CBR1 deficit in response to microplastics may represent a critical point that can lead to β-cell dysfunction.

Our study also showed changes in the abundance of proteins involved in fatty acid synthesis in the pancreas. A high dose of PET microplastics increased the abundance of fatty acid synthase (FASN) – a multifunctional enzyme responsible for de novo lipogenesis – and one of its catalytic domains, 3-oxoacyl-[acyl carrier protein] synthase (KAS), which plays a crucial role in the elongation of fatty acid chains by two carbon units during de novo lipogenesis [41]. FASN catalyzes the conversion of acetyl-CoA and malonyl-CoA to palmitate, which can be further elongated to stearate (C18:0) or modified to more complex fatty acids. Under physiological conditions, FASN is expressed at low levels in normal tissues [42], while its overexpression has been detected in pathological conditions, including insulin resistance, type 2 diabetes and various cancers [43]. However, despite the increased protein abundance of FASN, we found a decrease in its enzymatic activity. Such a discrepancy between protein abundance and enzymatic activity has been reported in other studies [44]. It may be explained by post-translational modifications, such as protein acetylation or ubiquitylation [45], which can impair catalytic function by decreasing enzyme stability, as well as by oxidative stress–induced disruptions in protein folding [46], triggered by PET microplastics. However, future mechanistic studies are required to clarify the mechanism by which microplastics influence post-translational modifications. At the same time, exposure to a high dose of PET microplastics resulted in a significant elevation of free fatty acids (FFA) level in the pancreas. This discrepancy– increased protein abundance but decreased enzymatic activity of FASN with simultaneous FFA accumulation – may indicate a dysfunction in the de novo lipogenesis pathway, in which upregulation of lipogenic proteins is not accompanied by functional activity. The elevated FFA level has been linked with pancreatic dysfunction. It has been reported that FFA can stimulate insulin secretion from β-cell, however prolonged exposure reduces insulin synthesis [47]. Moreover, chronic exposure to FFA leads to apoptosis of β-cell induced by endoplasmic reticulum (ER) stress and mitochondrial damage [48]. Therefore, we hypothesize that PET microplastics disrupt lipid metabolism in the pancreas by impairing de novo lipogenesis, leading to intracellular FFA accumulation and lipotoxicity. These processes can impair β-cell function and may contribute to the development of metabolic dysfunction.

The pancreas also has an essential exocrine role, producing digestive enzymes like amylase, lipase and trypsinogen, which are crucial for nutrients digestion and absorption [49]. There is evidence that trypsinogen is one of the most abundant pancreatic digestive enzymes [49] and low serum levels of trypsinogen in humans were strongly correlated with exocrine pancreatic insufficiency and diabetes mellitus, especially type 1 (T1D) [50]. Reduced trypsinogen levels were observed in patients with newly diagnosed T1D, in those with established T1D, as well as in individuals at high risk of developing T1D within 10 years [51–53]. Although our study measured trypsinogen levels in pancreatic tissue rather than serum, the results suggest that PET microplastics may contribute to the development of diseases associated with pancreatic insufficiency. These results suggest that PET microplastics may affect the efficiency of protein digestion in the duodenum by trypsin. It highlights a new mechanism by which microplastics may affect pancreas. However, further mechanistic studies are needed to confirm this hypothesis.

## Conclusion

In conclusion, our results demonstrated that PET microplastics affect both redox homeostasis and metabolic integrity of the pancreas in a dose-dependent manner. High doses of PET microplastics increase the abundance of TRX1 and PRDX5 – likely a protective response to PET-induced hyperglycemia and ROS overload. At the same time, exposure to high doses suppresses CBR1, which could exacerbate lipid peroxidation and β-cell apoptosis. Dysregulation of de novo lipogenesis is evidenced by the paradoxical upregulation and functional inhibition of FASN alongside FFA accumulation, suggesting lipotoxic stress. Finally, decreased trypsinogen abundance suggests impaired exocrine function with potential consequences for protein digestion and nutrient absorption. Taken together, these data reveal several converging mechanisms – oxidative stress, lipotoxicity, and exocrine insufficiency – by which PET microplastics may affect pancreatic physiology and contribute to metabolic disease.

## Supplementary Information

Supplementary Material 1.

## Data Availability

The raw acquisition LC–MS/MS data is deposited and freely accessible in MassIVE repository (dataset MSV000098442 at massive.ucsd.edu).
